# Uncovering mediational pathways behind racial and socioeconomic disparities in brain volumes: insights from the UK Biobank study

**DOI:** 10.1007/s11357-024-01371-1

**Published:** 2024-10-10

**Authors:** May A. Beydoun, Hind A. Beydoun, Marie T. Fanelli-Kuczmarski, Yi-Han Hu, Danielle Shaked, Jordan Weiss, Shari R. Waldstein, Lenore J. Launer, Michele K. Evans, Alan B. Zonderman

**Affiliations:** 1 Laboratory of Epidemiology and Population Sciences, National Institute On Aging, NIA/NIH/IRP, 251 Bayview Blvd., Suite 100, Room #: 04B118, Baltimore, MD 21224 USA; 2 Department of Research Programs, Fort Belvoir Community Hospital, Fort Belvoir, VA, 22060 USA; 3 Boston Veterans’ Affairs, Boston, MA 02130 USA; 4 Stanford Center On Longevity, Stanford University, Stanford, CA 94305 USA; 5 Department of Psychology, University of Maryland Baltimore County, Catonsville, MD 21250 USA

**Keywords:** Brain aging, Health disparities, Socioeconomic status, Lifestyle factors, Neurodegeneration

## Abstract

Mediation pathways explaining racial/ethnic and socioeconomic (SES) disparities in structural MRI markers of brain health remain underexplored. We examined racial/ethnic and SES disparities in sMRI markers and tested total, direct, and indirect effects through lifestyle, health-related, and cognition factors using a structural equations modeling approach among 36,184 UK Biobank participants aged 40–70 years at baseline assessment (47% men). Race (non-White vs. White) and lower SES-predicted poorer brain sMRI volumetric outcomes at follow-up, with racial/ethnic disparities in sMRI outcomes involving multiple pathways and SES playing a central role in those pathways. Mediational patterns differed across outcomes, with the SES-sMRI total effect being partially mediated for all outcomes. Over 20% of the total effect (TE) of race/ethnicity on WMH was explained by the indirect effect (IE), by a combination of different pathways going through SES, lifestyle, health-related, and cognition factors. This is in contrast to < 10% for total brain, gray matter (GM), white matter (WM), and frontal GM left/right. Another significant finding is that around 57% of the total effect for SES and the normalized white matter hyperintensity (WMH) was attributed to an indirect effect. This effect encompasses many pathways that involve lifestyle, health-related, and cognitive aspects. Aside from WMH, the percent of TE of SES mediated through various pathways ranged from ~ 5% for WM to > 15% up to 36% for most of the remaining sMRI outcomes, which are composed mainly of GM phenotypes. Race and SES were important determinants of brain volumetric outcomes, with partial mediation of racial/ethnic disparities through SES, lifestyle, health-related, and cognition factors.

**Supplementary Information:**

The online version contains supplementary material available at 10.1007/s11357-024-01371-1.

## Introduction

On a global scale, around 4.7% of elderly individuals are affected by dementia, a severe form of cognitive impairment that induces demand for caregiving related to everyday tasks. Each year, there are an additional 4.6–7.7 million new cases, resulting in an incidence rate of 3.5–10.6 per 1000 individuals per year [1]. Brain magnetic resonance imaging (MRI) offers an opportunity to examine in vivo brain changes and pathologies that may be related to cognitive impairment [2]. Some of these metrics, such as reduced frontal gray matter and hippocampal volumes and increased white matter hyperintensities or lesions, are considered early markers of AD, or part of the AD brain phenome [3].

Currently, there are no efficacious treatments available for either Alzheimer’s disease (AD) or all-cause dementia. Identifying and controlling changeable risk variables can enhance our capacity to prevent or delay their occurrence [4]. AD, particularly the sporadic type, has been ascribed to various modifiable and non-modifiable risk factors [4]. A little over 40% of all types of dementia can be attributed to various socio-economic, lifestyle, and health-related factors, which include but are not limited to low education in early life, mid-life obesity, and later-life social interactions [5].

Brain volumetric outcomes, particularly standardized hippocampal volumes and those of other subcortical and cortical regions of the brain, coupled with the volume of white matter lesions, play a significant role in understanding the cognitive health inequalities that are affected by race and socioeconomic status (SES) among older persons [6-8]. These brain-defined phenotypes can predict memory formation, cognitive function, and decline with age and can reflect overall neurological health [9-11]. They are important for evaluating and predicting cognitive decline and neurodegenerative illnesses like dementia. Recent research has identified substantial disparities in brain volume measurements among different racial and socioeconomic groups. In addition, there is a correlation between socioeconomic status (SES) and variations in brain structure. Individuals with lower SES are more prone to have larger volumes of white matter lesions and smaller volumes of the hippocampus and cortex [11-22].

Fully understanding these differences is essential as they indicate more extensive health inequalities in cognitive aging and the occurrence of diseases. Disparities in various brain-related phenotypes including volumetric outcomes can be attributed to socioeconomic variables, including healthcare accessibility, educational opportunities, environmental exposures, and stress levels [23-25]. These inequalities not only affect the health outcomes of individuals, but also have wider implications for public health policies and programs that aim to decrease cognitive decline and promote healthy aging [26].

Racial disparities persist in risk factors for dementia risk, particularly among non-Hispanic Black women in the United States and with respect to adiposity [27, 28]. Until recently, there has been a lack of research on the mechanisms that can explain the differences in brain health markers shown in structural MRI scans among different racial/ethnic and socioeconomic groups, especially in the UK population. Recent studies have mainly uncovered pathways for dementia outcomes and diffusion-weighted measures such as fractional anisotropy[8, 25].

In this investigation, we test racial/ethnic disparities in volumetric structural brain MRI metrics. We specifically examine whether such disparities are mediated through SES and test the role of other downstream factors in that process, including lifestyle, health-related, and cognitive function-related factors. We use data on ~ 45K UK Biobank middle-aged adults who have brain structural MRI measures of interest. We conduct structural equations models, assessing patterns of mediation of the association between race/ethnicity and sMRI volumetric outcomes through several pathways.

## Methods

### Data sources

The UK Biobank is an extensive biomedical database and research resource that has collected prospective data from more than 500,000 people between the ages of 37 and 73 years in the UK, with an initial recruitment between 2006 and 2010 [29]. The dataset comprises genetic data, phenotypic data, lifestyle information, imaging data, and biological samples. Participants completed self-administered questionnaires and face-to-face interviews at 22 assessment centers in England, Scotland, or Wales [29]. The questionnaires addressed topics such as sociodemographics, lifestyle exposures, early life exposures, psychological state, cognitive function, family history of illness, and medical history. The database serves as a potent tool for worldwide scientific study, facilitating extensive epidemiological investigations, pinpointing genetic and environmental risk factors, and fostering the creation of novel prevention and treatment approaches. The UK Biobank adheres to an ethical framework, ensuring the anonymity and permission of participants. The data is readily available to researchers globally, promoting international cooperation and optimizing the potential for significant breakthroughs. Approval was obtained from the North West Multi-Centre Research Ethics Committee [29].

### Study sample

**Fig. 1:**
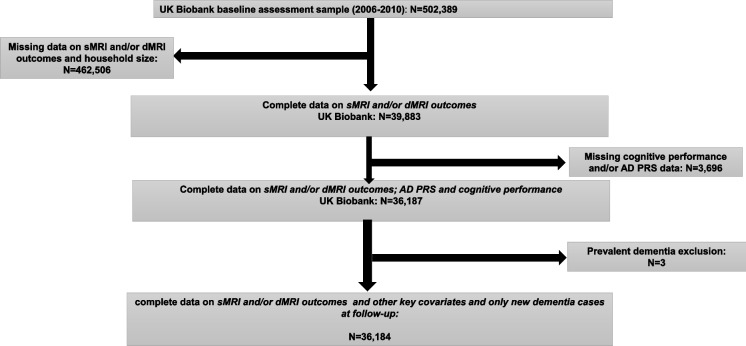
Participant flowchart

The original 502,399 UK Biobank participants were screened for the availability of brain MRI, AD polygenic risk score (PRS), cognitive performance score, household size, and other sociodemographics (Fig. 1). The final sample size consisted of 36,184 middle-aged and older adults. Of these, 47% were men, and the age range was 40–70 years. The final selected sample was also dementia-free at baseline [30]. Multiple imputation was carried out on all remaining potentially mediating socio-economic, lifestyle, and health-related variables (e.g., diet, physical activity, and cardiometabolic health).

### Brain MRI acquisition and processing

The UK Biobank study uses comprehensive brain MRI methods to provide detailed structural and functional insights into participants’ brains. This sub-study is an initiative that will include overtime over 100,000 participants aged 40–69 years. Specifically, the imaging visit completed MRI brain scans within sites around Reading, Newcastle, and Cheadle Manchester [31, 32]. Data is processed rigorously to ensure consistency and reliability. MRI data for the brain were acquired with a 3T Siemens Skyra scanner. For more details regarding the protocol, see http://www.fmrib.ox.ac.uk/ukbiobank/protocol/V4_23092014.pdf, and for the documentation, see http://biobank.ctsu.ox.ac.uk/crystal/docs/brain_mri.pdf, along with the following published work [33]. The study uses advanced image processing techniques to quantify brain volumetric measurements. Functional MRI data examines connectivity patterns between brain regions during resting-state conditions. Diffusion imaging assesses white matter integrity. By utilizing a 256-cm superior–inferior field of view, scans from the upper part of the head down to the neck were carried out [31, 32, 34].

The UK Biobank utilized global tissue volumes (sMRI) and white matter tract-averaged water molecular diffusion indices (dMRI) to generate imaging-derived phenotypes (IDPs) [35]. The study utilized data on around 43,000 brain MRI structural phenotypes that were made available in November 2022 [35]. The chosen imaging characteristics were linked to poorer cognitive function and deterioration, encompassing overall volumes of white and grey matter, volumes of frontal grey matter on both sides, as well as volumes of sub-cortical structures, including the hippocampus. Intracranial volume or ICV was incorporated into the definition of WMH phenotype (i.e., WMH as % ICV, Log_e_-transformed). It was also included in models with subcortical volumes as the outcome as a potential confounder [36].

### Race/ethnicity

Race/ethnicity was operationalized by re-grouping a detailed version of ethnic background into White, Black, South Asian, and Others for descriptive purposes. This measure was further modified to reflect minority status and was re-coded as non-White vs. White.

### Mediators

#### Socio-economic status (SES)

SES was determined by integrating measures of income, education, and the Townsend deprivation index (TDI). The baseline education was assessed using a 3-point scale (0, low, combining “CSEs/Equivalent,” “NVQ/HND/HNC/Equivalent,” and “Other professional qual”; 1, intermediate, combining “O Levels/GCSEs/Equivalent” and “A/AS Levels Equivalent’; 2, higher level or “College/University”), while the pre-tax total family income was categorized using a 5-point scale (1 = “£18,000,” 2 = “£18,000–£29,999,” 3 = “£30,000–£51,999,” 4 = “52,000–£100,000,” and 5 = “ > £100,000”) [8, 25]. The TDI was calculated using data from the National Census, including household overcrowding, car ownership, owner–occupier status, and unemployment. The original coding of higher TDI was designed to represent lower SES, but in this study, the TDI was multiplied by − 1, which resulted in SES being reflected by a higher TDI score (reverse coded). A summary score for SES was generated by calculating the mean of the inverted TDI, education, and income *z*-scores.

#### Lifestyle and health-related factors

We analyzed six lifestyle factors: smoking, alcohol, physical activity, diet quality, nutritional markers, and social support [8, 25]. These factors were operationalized using varying numbers of items. The study also examined poor cardio-metabolic and general health using four measured variables: body mass index, allostatic load, co-morbidity index, and self-rated health [8, 25]. Poor cognitive performance was assessed using three items from two cognitive tests [8, 25]. Further details on these mediators’ operationalization are provided in Supplementary Method 2.

### Exogenous covariates

Covariates that were considered exogenous to the SEM included sociodemographic factors such as age, sex, and household size. Furthermore, and only for the final equation in the SEM, AD Polygenic Risk Score (PRS, see Supplementary Method 2) and time between baseline assessment and the imaging visit in days were included along with an inverse mills ratio to correct for selection bias. Finally, to correct for head size, ICV was added to the final outcome equation when the outcomes were subcortical volumes.

### Statistical methods

Analyses were implemented with Stata 17.0 (StataCorp, College Station, TX) [37]. Type I error was set at 0.05. Data aside from exogenous variables, cognitive performance, and *sMRI* outcomes were imputed using chained Eqs. (5 imputations, 10 iterations), given that they generally had less than 10% missing data individually, after selecting the largest eligible sample [38]. This was carried out in Stata using *mi impute*, *mi passive*, *mi register/unregister*, and *mi estimate* commands among others, applying various types of models to each level of measurement (mainly linear, ordinal logistic, and multinomial logit). *mi extract* and *mi xeq* were also utilized to extract each imputation for the purpose of obtaining non-linear combinations of parameters and then combining all 5 imputation findings by using Rubin’s rule. Detailed Stata script is provided on the github at baydounm/UKB-paper5-RaceSESsMRI (github.com). Both categorical and continuous sample characteristics were compared by race/ethnicity (non-White vs. White), using a series of OLS linear and multinomial logit models, whereby racial minority status was the only predictor. A single two-sided *p*-value is presented as obtained from the OLS linear models, whereas several *p*-values are presented from the multinomial logit models taking the largest category from each categorical outcome as the referent. The non-White vs. White contrast was tested for all *sMRI* outcomes (both cortical and sub-cortical volumetric outcomes) in a series of OLS multiple linear regression models, adjusted in an incremental manner for other exogenous variables, SES, lifestyle factors, health factors, and cognition, in three separate models. Changes in the racial minority point estimate across models were assessed using a cutoff of 10% of appreciable changes between reduced and adjusted models.

Incorporating sociodemographic data as exogenous variables and endogenous variables such as SES, lifestyle factors, health, and COGN variables, the study employed structural equation models (SEM) to assess mediation. SES, health-related and lifestyle variables, and poor cognition were predicted by RACE_ETHN in relation to sMRI cortical and sub-cortical volumes, which were the final outcomes of interest. It is hypothesized among others that hippocampal volumes would be smaller and WMH would be larger with poorer cognition Importantly, pathways between endogenous variables and between RACE_ETHN and each endogenous variable were included in this SEM model (Fig. 1). Given the large sample size available for this analysis, only effects detected with *p*-values < 0.001 were interpreted as statistically significant.

Next, the direct and indirect effects (DE and IE) of race/ethnicity were decomposed starting from the total effect (TE) of race/ethnicity and other endogenous variables on sMRI outcomes. The IE might be a mix of several different pathways. However, for ease of interpretation, we plotted the ROIs for subcortical volumes on a conventional MNI brain to illustrate the key patterns of relationships with respect to TE, DE, and IE and their statistical significance in these models. The FSLeyes program (URL: https://fsl.fmrib.ox.ac.uk/fsl/¬fslwiki/FSLeyes) was used to visualize the findings of the sub-cortical SEM pattern and pathway. Using R version 4.2.2 (https://www.r-project.org/), TE, DE, and IE were additionally visualized through the use of heat maps for subcortical volumes among others to show the statistical significance and direction of association.

Furthermore, a number of IE were of primary interest for a subset of sMRI outcomes (total brain, WMH as % ICV (Loge-transformed), and hippocampus volumes (L/R)). These IE were estimated by multiplying and adding effects starting from the race/ethnicity contrast and ending in the final sMRI volumetric outcomes through each of the serial sets of mediators [39]. For every lifestyle component, six distinct pathways were found to be of relevance and were qualitatively assessed for statistical significance at a type I error of 0.05, which was further corrected for multiple testing to a threshold of 0.001.

Pathway A: RACE_ETHN → SES → sMRI; Pathway B: RACE_ETHN → SES → LIFESTYLE → sMRI; Pathway C: RACE_ETHN → SES → LIFESTYLE → HEALTH → sMRI; Pathway D: RACE_ETHN → SES → LIFESTYLE → HEALTH → POOR COGNITIVE PERFORMANCE (COGN) → sMRI; Pathway E: RACE_ETHN→SES→COGN→sMRI; Pathway F: RACE_ETHN→SES→LIFESTYLE→COGN→sMRI. It is hypothesized that Pathway D is responsible for the racial/ethnic disparities in those chosen sMRI results. A proportion mediated was also computed out of the TE and out of the total IE. While the proportion mediated does not necessarily carry a set criterion, we have used a threshold of 10% for appreciable mediated effect out of the TE. By including an inverse mills ratio, which is a function of the conditional probability of being selected given a set of sociodemographic variables, namely baseline age, sex, and race/ethnicity, OLS multiple linear regression models accounted for sample selectivity using a 2-stage Heckman selection approach, as did the SEM models [40].

## Results

Non-White participants in this study were younger than their White counterparts. They were also represented proportionately by fewer female participants and had higher educational attainment, but greater socioeconomic deprivation as measured by TDI (Table 1). White adults on the other hand were more likely than their non-White counterparts to be former smokers and to consume alcohol frequently. Diet quality was on average better among non-White adults, even though White adults had a better profile of nutritional biomarkers. Social support was found to be more deficient among non-White adults, who also had poorer health, and scored lower on 3 cognitive test scores. The crude association between racial minority status and sMRI outcomes indicated smaller brain volumes and ICV. Also based on unadjusted models, subcortical volumes were smaller among non-White adults vs. White adults, with few exceptions (left accumbens, right pallidum) for which no difference was detected. AD PRS was indicative of lower AD risk among non-White adults.

**Table 1:** Study sample characteristics by race/ethnicity: the UK Biobank 2006–2021

Study sample characteristics	Overall	White	Non-White	*p*_race_
	***N*** = 36,184	***N*** = 35,084	***N*** = 1100	
**Sociodemographic**				
** Baseline age, y**	55.57 ± 0.04	55.66 ± 0.04	52.49 ± 0.23	< 0.001
** Sex, % female**	53.2	53.3	49.9	0.027
**Race/ethnicity**				
** White**	97.0	100.0	0.0	__
** Black**	0.6	0.0	19.5	
** South Asian**	1.0	0.0	32.0	
** Other**	1.5	0.0	48.5	
** Household size**	2.54 ± 0.01	2.53 ± 0.01	2.97 ± 0.05	< 0.001
**Socio-economic status**				
**Education**				
Low	16.0	16.0	15.8	0.012
Intermediate	35.2	35.6	24.4	< 0.001
High	48.8	48.4	59.8	__
** Income, range 1–5**	2.97 ± 0.01	2.97 ± 0.01	2.97 ± 0.04	0.87
** Townsend Deprivation Index**	− 1.89 ± 0.01	− 1.93 ± 0.01	− 0.384 ± 0.099	< 0.001
** SES *z*-score**	− 0.0066 ± 0.0034	− 0.0025 ± 0.0034	− 0.137 ± 0.024	< 0.001
**Lifestyle factors**				
**Smoking**				
**Smoking status**				
Never	76.6	76.4	80.9	__
Former	17.3	17.5	11.3	< 0.001
Current	6.1	6.0	7.8	0.082
Environmental tobacco smoke	0.652 ± 0.022	0.649 ± 0.022	0.738 ± 0.109	0.48
Pack-years of tobacco smoke	0.107 ± 0.001	0.108 ± 0.001	0.085 ± 0.006	0.003
** SMOKING *z*-score**	+ 0.022 ± 0.004	+ 0.023 ± 0.004	− 0.0196 ± 0.021	0.054
** Alcohol consumption**	3.330 ± 0.007	3.362 ± 0.007	2.296 ± 0.050	< 0.001
**Alcohol consumption frequency, range 0–5**				
** ALCOHOL *z*-score**	− 0.0000 ± 0.0053	+ 0.0234 ± 0.005	− 0.7461 ± 0.0358	< 0.001
**Physical activity, PA**				
** PA, Met.min.wk**^**−1**^	1,865 ± 13	1,868 ± 13	1763 ± 70	0.15
** PA *z*-score**	− 0.0000 ± 0.0053	+ 0.0013 ± 0.0053	− 0.0427 ± 0.0293	0.15
**Diet quality**				
** HDI**	5.235 ± 0.007	5.231 ± 0.008	5.338 ± 0.043	0.016
** DIET *z*-score**	0.0000 ± 0.0053	− 0.00223 ± 0.0053	+ 0.0714 ± 0.0300	0.016
**Nutritional biomarkers**				
** 25-hydroxyvitamin D**	49.7 ± 0.12	50.2 ± 0.12	34.40 ± 0.56	< 0.001
** Red cell distribution width**	13.404 ± 0.005	13.396 ± 0.005	13.650 ± 0.038	< 0.001
** NUTR *z*-score**	− 0.0007 ± 0.0038	+ 0.0150 ± 0.0038	− 0.5025 ± 0.0249	< 0.001
**Social support**				
** “How often do you visit friends or family or have them visit you?”**	5.170 ± 0.006	5.184 ± 0.006	4.747 ± 0.034	< 0.001
** “How often are you able to confide in someone close to you?”**	1.085 ± 0.005	1.088 ± 0.005	0.975 ± 0.026	< 0.001
** “Which of the following do you attend once a week or more often?”**	3.686 ± 0.010	3.701 ± 0.010	3.226 ± 0.060	< 0.001
** SS *z*-score**	− 0.0003 ± 0.0033	+ 0.0077 ± 0.0033	− 0.2579 ± 0.020	< 0.001
**Cardio-metabolic and general health-related factors**				
** Body mass index, kg.m**^**−1**^	26.51 ± 0.02	26.51 ± 0.022	26.42 ± 0.12	0.47
** Allostatic load**	1.687 ± 0.008	1.686 ± 0.009	1.715 ± 0.041	0.49
** Co-morbidity index**	1.570 ± 0.009	1.571 ± 0.009	1.538 ± 0.050	0.51
** Self-rated health, range 1–4**	1.939 ± 0.003	1.925 ± 0.004	2.060 ± 0.022	< 0.001
** HEALTH *z*-score**	0.0011 ± 0.0035	− 0.0003 ± 0.0036	+ 0.0453 ± 0.0200	0.025
**Cognitive performance**				
** Reaction time**	6.2724 ± 0.0009	6.271 ± 0.00091	6.306 ± 0.00595	< 0.001
** Pairs matching, errors**	0.5733 ± 0.0036	0.5670 ± 0.0037	0.7748 ± 0.0214	< 0.001
** Pairs matching, time to complete**	5.2367 ± 0.0018	5.2330 ± 0.0018	5.356 ± 0.0123	< 0.001
** COGN *z*-score**	− 0.2986 ± 0.0064	− 0.3129 ± 0.0064	0.15773 ± 0.0426	< 0.001
**sMRI outcomes, mm**^**3**^				
ICV	1,547,757 ± 809	1,549,844 ± 811	1,488,551 ± 5,875	< 0.001
Total brain volume	1,158,573 ± 585	1,159,990 ± 593	1,113,375 ± 3,298	< 0.001
Total GM	613,940 ± 293	614,686 ± 297	590,137 ± 1,681	< 0.001
Total WM	544,633 ± 323	545,304 ± 328	523,239 ± 1,783	< 0.001
Frontal GM, left brain	75,814 ± 42	75,891 ± 42.8	73,359 ± 237	< 0.001
Frontal GM, right brain	75,474 ± 42	75,552 ± 42	72,997 ± 236	< 0.001
White matter hyperintensity, WMH, % ICV, Log_e_-transformed	− 1.622 ± 0.005	− 1.616 ± 0.005	− 1.780 ± 0.027	< 0.001
***Subcortical volumes***				
Accumbens, left	490.9 ± 0.6	491.0 ± 0.647	489.0 ± 3.5	0.58
Accumbens, right	384.9 ± 0.6	385.1 ± 0.6	377.5 ± 3.1	0.026
Amygdala, left	1261.8 ± 1.3	1262.9 ± 1.3	1225.6 ± 7.3	< 0.001
Amygdala, right	1225.9 ± 1.4	1227.2 ± 1.46	1186.0 ± 8.0	< 0.001
Caudate, left	3375.2 ± 2.2	3378.2 ± 2.3	3280.0 ± 12.4	< 0.001
Caudate, right	3558.1 ± 2.3	3561.4 ± 2.4	3451.2 ± 12.9	< 0.001
Hippocampus, left	3769.3 ± 2.5	3770.3 ± 2.6	3739.0 ± 13.7	0.035
Hippocampus, right	3884.1 ± 2.6	3885.4 ± 2.7	3843.7 ± 14.2	0.006
Pallidum, left	1753.2 ± 1.3	1753.8 ± 1.3	1731.9 ± 7.4	0.004
Pallidum, right	1797.0 ± 1.3	1797.2 ± 1.3	1790.4 ± 7.6	0.37
Putamen, left	4752.4 ± 3.2	4754.6 ± 3.2	1731.9 ± 7.4	< 0.001
Putamen, right	4810.1 ± 3.1	4812.8 ± 3.2	1790.4 ± 7.6	< 0.001
Thalamus, left	7734.3 ± 4.0	7738.4 ± 4.1	7602.8 ± 22.7	< 0.001
Thalamus, right	7543.0 ± 3.9	7547.6 ± 4.0	7396.2 ± 21.7	< 0.001
**AD PRS**				
Mean ± SE	0.0406 ± 0.0052	0.043 ± 0.005	− 0.045 ± 0.031	0.004
T1	33.3	33.3	36.0	__
T2	33.3	33.3	33.3	0.27
T3	33.3	33.4	30.7	0.030
** Follow-up time, days**	3289 ± 3	3291 ± 3	3245 ± 19	0.019

*AD* Alzheimer’s disease, *ALCOHOL* alcohol consumption *z*-score, *COGN* poor cognitive performance *z*-score, *DIET*, diet quality *z*-score; *dMRI*, diffusion-weighted magnetic resonance imaging; *FA*, fractional anisotropy; *GM*, gray matter; *HDI*, Healthy Diet Index; *HEALTH*, poor cardio-metabolic and general health *z*-score; *ICV*, intracranial volume; *PA*, physical activity *z*-score; *PRS*, polygenic risk score; *MD*, mean diffusivity; *NUTR*, nutritional biomarker *z*-score; *SES*, socio-economic status *z*-score; *SMOKING*, smoking *z*-score; *sMRI*, structural magnetic resonance imaging; *SS*, social support *z*-score; *T1*, first tertile; *T2*, second tertile; *T3*, third tertile; *WM*, white matter; *WMH*, white matter hyperintensity

Values are means ± SE or percentages in multiple imputed data

Table 2 shows results for three OLS multivariable linear regression analyses (M1-3) with race/ethnicity as the main predictor for volumetric outcomes. Each analysis incrementally adjusted for potential mediating variables. As was the case for crude associations, most analyses indicated smaller volumetric outcomes among non-White adults vs. White adults and greater WMH as % ICV (Log_e_-transformed) among non-White adults vs. White adults. Examining the results between M1 and M3 suggests that SES combined with other upstream mediating factors explained between 5 and 40–50% of the sociodemographic and AD-PRS adjusted differences in M1, depending on the volumetric outcome. Most notably, the racial difference in the left thalamus volume of − 48 ± 17 mm^3^ (*p* < 0.010) was attenuated to − 33 ± 17 mm^3^ (*p* > 0.05), after adjustment for SES, lifestyle, health-related, and cognitive performance co-predictors. While a similar attenuation was observed in the right thalamus, racial difference in that sub-cortical volume remained statistically significant in the fully adjusted model 3. SES was sufficient to attenuate the association between race and right amygdala volume by > 15%, while other upstream factors attenuated the effect further. Similar patterns of attenuation were noted for bi-lateral putamen and accumbens volumes among other subcortical structures. A combination of SES, lifestyle, health-related, and cognitive performance variables attenuated the sociodemographic and AD PRS-adjusted race effect on total brain volume by < 10%, while 18% of the race effect of increased WMH (as% ICV, Log_e_-transformed) among non-White adults vs. White adults was explained by those same factors.

**Table 2:** Racial/ethnic disparities in sMRI volumetric outcomes, *N* = 36,184: the UK Biobank 2006–2021

sMRI volumetric outcomes	*β* ± SE, *p*	(95% CI)
Total brain volume		
M1	− 66,582 ± 2,698***	(− 71,870, − 61,294)
M2	− 63,772 ± 2,690***	(− 69,044, − 58,500)
M3	− 61,442 ± 2,735***	(− 66,803, − 56,081)
Total GM		
M1	− 36,729 ± 1,370***	(− 39,414, − 34,044)
M2	− 35,226 ± 1,366***	(− 37,903, − 32,549)
M3	− 34,418 ± 1,386 ***	(− 37,135, − 31,701)
Total WM		
M1	− 29,853 ± 1,514 ***	(− 32,820, − 26,886)
M2	− 28,546 ± 1,512***	(− 31,510, − 25,582)
M3	− 27,024 ± 1538***	(− 30,038, − 24,010)
Frontal GM, left brain		
M1	− 4,158 ± 208***	(− 4,566, − 3,750)
M2	− 3,985 ± 208***	(− 4,393, − 3,577)
M3	− 3,947 ± 211***	(− 4,361, − 3,533)
Frontal GM, right brain		
M1	− 4,122 ± 208***	(− 4,530, − 3,714)
M2	− 3,951 ± 207 ***	(− 4,357, − 3,545)
M3	− 3,911 ± 211***	(− 4,325, − 3,497)
White matter hyperintensity, WMH, % ICV, Log_e_-transformed		
M1	0.0698 ± 0.0261**	(0.019, 0.121)
M2	0.0564 ± 0.0261*	(0.005, 0.108)
M3	0.0555 ± 0.0263*	(0.004, 0.107)
***Subcortical volumes***		
Accumbens, left		
M1	− 7.31 ± 3.30*	(− 13.78, − 0.84)
M2	− 7.41 ± 3.31*	(− 13.90, − 0.92)
M3	− 6.10 ± 3.36	(− 12.69, 0.49)
Accumbens, right		
M1	− 17.24 ± 3.05***	(− 23.22, − 11.26)
M2	− 17.01 ± 3.05***	(− 22.99, − 11.03)
M3	− 16.39 ± 3.10***	(− 22.47, − 10.31)
Amygdala, left		
M1	− 10.47 ± 7.14	(− 24.46, 3.52)
M2	− 9.01 ± 7.15	(− 23.02, 5.00)
M3	− 7.61 ± 7.28	(− 21.88, 6.66)
Amygdala, right		
M1	− 16.21 ± 8.03*	(− 31.95, − 0.47)
M2	− 14.73 ± 8.04	(− 30.49, 1.03)
M3	− 10.79 ± 8.19	(− 26.84, 5.26)
Caudate, left		
M1	− 16.02 ± 10.87	(− 37.33, 5.29)
M2	− 13.39 ± 10.89	(− 34.73, 7.95)
M3	− 10.68 ± 11.10	(− 32.44, 11.08)
Caudate, right		
M1	− 17.92 ± 11.57	(− 40.60, 4.76)
M2	− 15.06 ± 11.59	(− 37.78, 7.66)
M3	− 9.74 ± 11.81	(− 32.89, 13.41)
Hippocampus, left		
M1	− 23.79 ± 13.53	(− 50.31, 2.73)
M2	− 20.43 ± 13.54	(− 46.97, 6.11)
M3	− 13.54 ± 13.79	(− 40.57, 13.49)
Hippocampus, right		
M1	− 22.43 ± 13.73	(− 49.34, 4.48)
M2	− 19.07 ± 13.74	(− 46.00, 7.86)
M3	− 15.44 ± 14.00	(− 42.88, 12.00)
Pallidum, left		
M1	9.84 ± 6.80	(− 3.49, 23.17)
M2	11.37 ± 6.80	(− 1.96, 24.70)
M3	14.14 ± 6.92*	(0.58, 27.70)
Pallidum, right		
M1	18.26 ± 6.77**	(4.99, 31.53)
M2	20.16 ± 6.77**	(6.89, 33.43)
M3	23.93 ± 6.88**	(10.45, 37.41)
Putamen, left		
M1	− 39.12 ± 14.73**	(− 67.99, − 10.25)
M2	− 37.10 ± 14.75*	(− 66.01, − 8.19)
M3	− 28.94 ± 15.00	(− 58.34, 0.46)
Putamen, right		
M1	− 43.68 ± 14.37**	(− 71.85, − 15.51)
M2	− 41.25 ± 14.39**	(− 69.45, − 13.05)
M3	− 34.36 ± 14.65*	(− 63.07, − 5.65)
Thalamus, left		
M1	− 47.59 ± 16.95**	(− 80.81, − 14.37)
M2	− 44.93 ± 16.95**	(− 78.15, − 11.71)
M3	− 33.35 ± 17.22	(− 67.10, 0.40)
Thalamus, right		
M1	− 62.08 ± 16.00***	(− 93.44, − 30.72)
M2	− 59.17 ± 16.00***	(− 90.53, − 27.81)
M3	− 50.51 ± 16.25**	(− 82.36, − 18.66)

*AD*, Alzheimer’s disease; *ALCOHOL*, alcohol consumption *z*-score; *COGN*, poor cognitive performance *z*-score; *DIET*, diet quality *z*-score; *GM*, gray matter; *HDI*, Healthy Diet Index; *HEALTH*, poor cardio-metabolic and general health *z*-score; *ICV*, intracranial volume; *PA*, physical activity *z*-score; *PRS*, polygenic risk score; *NUTR*, nutritional biomarker *z*-score; *SES*, socio-economic status *z*-score; *SMOKING*, smoking *z*-score; *sMRI*, structural magnetic resonance imaging; *SS*, social support *z*-score; *WM*, white matter; *WMH*, white matter hyperintensity

*p < 0.05, **p < 0.010, ***p < 0.001

M1: adjusted for age, sex, AD PRS, household size, follow-up time (days), and inverse mills ratio. ICV adjusted for in the case of subcortical volumes. M2: M1 further adjusted for SES, M3: M2 further adjusted for DIET, SMOKING, ALCOHOL, NUTR, SS, HEALTH, and COGN *z*-scores

**Fig. 2:**
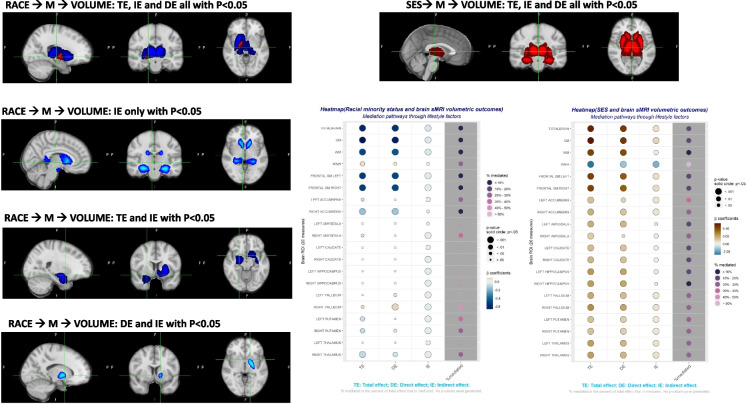
Main findings from SEM models on standard brain images and heat maps for racial minority status (non-White vs. White) and SES total, indirect, and direct effects on sMRI outcomes^a,b^. Abbreviations: DE, direct effect; IE, indirect effect; SEM, structural equations model; SES, socio-economic status; TE, total effect; ^a^patterns plotted on standard brain images pertain only to subcortical structures and are based on the statistical significance of TE, IE, and DE. Dark blue color is for significant TE reflecting an inverse association with the subcortical structure. The light blue color is used when TE is non-significant, but IE is significant reflecting an inverse association with the subcortical structure through a series of mediators. Dark red is for a positive association based on a significant, positive TE. Brain image visualization used a standard MNI 152 brain template and FSLEYES software: https://fsl.fmrib.ox.ac.uk/fsl/fslwiki/FSLeyes. ^b^Heat map shows the associations of race and SES with all sMRI outcomes, focusing on standardized associations (TE, IE, and DE) and percent-mediated. For TE, DE, and IE, blueish colors are for inverse associations and brownish colors are for positive associations. The size of the circle pertains to *p*-values associated with TE, IE, and DE. Pink/purple colors are for proportion-mediated, irrespective of the direction of the IE or TE. However, those were left empty when *p*-values associated with TE were > 0.05. Heatmaps were generated using R Software

**Fig. 3:**
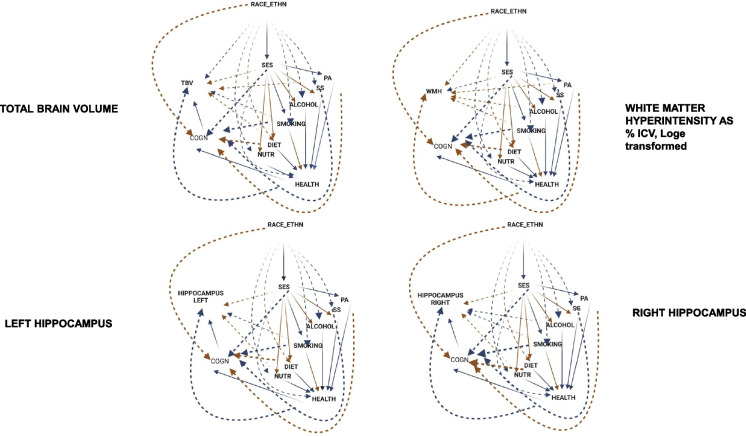
SEM findings for key sMRI outcomes: TBV, WMH, left and right hippocampal volumes^a,b^. Abbreviations: ALCOHOL, alcohol consumption *z*-score; COGN, poor cognitive performance *z*-score; DIET, diet quality *z*-score; HEALTH, poor cardio-metabolic and general health *z*-score; ICV, intracranial volume; PA, physical activity *z*-score; NUTR, nutritional biomarker *z*-score; SD, standard deviation; SEM, structural equation model; SES, socio-economic status *z*-score; SMOKING, smoking *z*-score; sMRI, structural magnetic resonance imaging; SS, social support *z*-score; WM, white matter; WMH, white matter hyperintensity. ^a^Arrows indicate statistically significant direct effects from SEM models. Blue arrows stand for inverse relationships (*β* < 0, *p* < 0.05), red arrow stands for positive relationships (*β* > 0, *p* < 0.05), solid lines are for direct effects that are part of the hypothesized pathway; dashed lines are direct effects outside the hypothesized pathway. ^b^See “Methods” section for a full list of exogenous variables entered into the SEM model. ICV was only included for subcortical structures

The findings from the structural equations modeling analysis, which examined the relationship between race, socioeconomic status (SES), and volumetric outcomes, are displayed in Tables 3, 4, and Supplementary Tables 2–3. Additionally, the results are visually represented in Figs. 2 and 3.

**Table 3:** Racial/ethnic disparities in sMRI volumetric outcomes: total, direct, and indirect effects through SES, lifestyle, health and cognition factors: the UK Biobank 2006–2021^a,b^

	Total effect			Direct Effect			Indirect effect			Percent-mediated	SD value
	*β*	*SE*	*p*	*β*	*SE*	*p*	*β*	*SE*	*p*		
***Brain volumes, mm***^***3***^											
Total brain volume	** − 61,442**	**2697**	** < 1.e-20**	** − 61,442**	**2731**	** < 1.e-20**	** − 5169**	**636**	**4.6e-16**	7.8	111,279
Total GM	** − 36,754**	**1370**	** < 1.e-20**	** − 34,418**	**1379**	** < 1.e-20**	** − 2336**	**335**	**3.2e-12**	6.4	55,735
Total WM	** − 29,857**	**1514**	** < 1.e-20**	** − 27,024**	**1536**	** < 1.e-20**	** − 2833**	**343**	**1.4e-16**	9.5	61,441
***Cortical frontal volumes, mm***^***3***^											
Frontal GM, left brain	** − 4161**	**208**	** < 1.e-20**	** − 3947**	**211**	** < 1.e-20**	** − 214**	**48**	**8.6e-06**	5.1	7989
Frontal GM, right brain	** − 4125**	**207**	** < 1.e-20**	** − 3911**	**210**	** < 1.e-20**	** − 214**	**48**	**8.1e-06**	5.2	7989
***White matter hyperintensity, WMH, % ICV, Log***_***e***_***-transformed***	**0.071**	**0.026**	**0.0068**	**0.055**	**0.026**	**0.034**	**0.015**	**0.007**	**0.024**	21.3	0.951
***Subcortical volumes***											
Accumbens, left	** − 8.2**	**3.3**	**0.013**	− 6.1	3.4	0.069	** − 2.1**	**0.70**	**0.003**	25.6	114
Accumbens, right	** − 18.1**	**3.0**	**3.2e-09**	− 16.4	3.1	1.3e-07	** − 1.7**	**0.7**	**0.011**	9.2	114
Amygdala, left	− 11.3	7.1	0.11	− 7.6	7.3	0.30	** − 3.7**	**1.44**	**0.011**	32.9	247
Amygdala, right	** − 16.2**	**8.2**	**0.043**	− 10.8	8.2	0.19	** − 5.4**	**1.6**	**0.001**	33.6	266
Caudate, left	− 16.6	10.9	0.13	− 10.7	11.1	0.33	** − 5.9**	**2.2**	**0.008**	35.7	418
Caudate, right	− 18.7	11.6	0.11	− 9.8	11.8	0.41	** − 8.9**	**2.4**	**0.0002**	47.8	438
Hippocampus, left	− 26.4	13.5	0.051	− 13.5	13.8	0.33	** − 12.8**	**2.8**	**6.2e-06**	48.6	476
Hippocampus, right	− 24.8	13.7	0.07	− 15.4	14.0	0.27	** − 9.4**	**2.9**	**0.0010**	37.8	494
Pallidum, left	+ 8.9	6.8	0.19	+ 14.1	**6.9**	**0.041**	** − 5.3**	**1.4**	**0.0002**	− 59.5	247
Pallidum, right	** + 16.9**	**6.8**	**0.012**	** + 23.9**	**6.9**	**0.005**	** − 7.1**	**1.5**	**1.5e-06**	− 42.0	247
Putamen, left	** − 41.6**	**14.7**	**0.005**	− 28.9	15.0	0.053	** − 12.7**	**3.1**	**0.00004**	30.4	608
Putamen, right	** − 45.5**	**14.4**	**0.002**	** − 34.4**	**14.6**	**0.018**	** − 11.1**	**3.0**	**0.0002**	24.4	590
Thalamus, left	− 15.4	13.4	0.25	− 33.3	17.2	0.052	** − 8.0**	**3.1**	**0.0089**	33.3	760
Thalamus, right	** − 67.5**	**16.0**	**0.00003**	** − 50.5**	**16.2**	**0.0018**	** − 17.0**	**3.6**	**2.0e-06**	25.2	741

Bolded values represent statistically significant estimates at type I error of 0.05

*AD*, Alzheimer’s disease; *ALCOHOL*, alcohol consumption *z*-score; *COGN*, poor cognitive performance *z*-score; *DIET*, diet quality *z*-score; *GM*, gray matter; *HDI*, Healthy Diet Index; *HEALTH*, poor cardio-metabolic and general health *z*-score; *ICV*, intracranial volume; *PA*, physical activity *z*-score; *PRS*, polygenic risk score; *NUTR*, nutritional biomarker *z*-score; *SD*, standard deviation; *SES*, socio-economic status *z*-score; *SMOKING*, smoking *z*-score; *sMRI*, structural magnetic resonance imaging; *SS*, social support *z*-score; *WM*, white matter; *WMH*, white matter hyperintensity

^a^Values are total, indirect, and direct effects of race with their associated standard errors and *p*-values, percent of total effect that is mediated and standard deviation value of each outcome. SEM models used are summarized in Supplementary Fig. 1. Selected numerical findings of key path coefficients are presented in Supplementary Table 2 and 3 and illustrated in Fig. 3. Standardized total, indirect, and direct effects are further presented in Fig. 2 (heatmap)

^b^See “Methods” section for a full list of exogenous variables entered into the SEM model. ICV was only included for subcortical structures

**Table 4:** Socio-economic disparities in sMRI volumetric outcomes: total, direct, and indirect effects through lifestyle, health and cognition factors: the UK Biobank 2006–2021

	Total effect			Direct Effect			Indirect effect			Percent-mediated	SD value
	*β*	*SE*	*p*	*β*	*SE*	*p*	*β*	*SE*	*p*		
***Brain volumes, mm***^***3***^											
Total brain volume	**13,991**	**726**	** < 1.e-20**	**12,365**	**754**	** < 1.e-20**	**1525**	**217**	**2.1e-12**	10.0	111,279
Total GM	**7493**	**369**	** < 1.e-20**	**6348**	**382**	** < 1.e-20**	**1144**	**112**	**1.8e-24**	15.3	55,735
Total WM	**6499**	**408**	** < 1.e-20**	**6117**	**424**	** < 1.e-20**	**382**	**121**	**0.001628**	5.9	61,441
***Cortical frontal volumes, mm***^***3***^											
Frontal GM, Left Brain	**863**	**56**	** < 1.e-20**	**742**	**58**	**4.2e-37**	**121**	**17**	**7.5e-13**	14.0	7989
Frontal GM, Right Brain	**856**	**56**	** < 1.e-20**	**721**	**58**	**4.2e-37**	**134**	**17**	**1.6e-15**	15.7	7989
***White matter hyperintensity, WMH, % ICV, Log***_***e***_***-transformed***	** − 0.067**	**0.007**	**2.5e-21**	** − 0.028**	**0.007**	**0.00007**	** − 0.038**	**0.002**	** < 1.e-20**	57.0	0.951
***Subcortical volumes***											
Accumbens, left	**3.8**	**0.9**	**0.00002**	**2.4**	**0.9**	**0.00894**	**1.4**	**0.3**	**2.9e-07**	36.0	114
Accumbens, right	**5.0**	**0.8**	**1.4e-09**	**3.6**	**0.9**	**0.0025**	**1.4**	**0.3**	**1.3e-08**	28.0	114
Amygdala, left	**11.2**	**1.9**	**7.6e-09**	**9.9**	**1.9**	**7.6e-09**	**1.3**	**0.6**	**0.020935**	11.6	247
Amygdala, right	**7.2**	**2.2**	**0.0009**	**5.4**	**1.8**	**0.017**	**1.8**	**0.6**	**0.003647**	25.5	266
Caudate, left	**15.4**	**2.9**	**1.5e-07**	**13.2**	**3.1**	**0.00015**	**2.2**	**0.9**	**0.009679**	14.4	418
Caudate, right	**17.4**	**3.1**	**2.9e-08**	**14.6**	**3.3**	**6.8e-06**	**2.7**	**0.9**	**0.002561**	15.9	438
Hippocampus, left	**28.2**	**3.7**	**1.3e-13**	**25.2**	**3.9**	**5.8e-11**	**2.2**	**1.1**	**0.001145**	12.4	476
Hippocampus, right	**27.5**	**3.7**	**1.3e-13**	**25.2**	**3.9**	**5.8e-11**	**2.2**	**1.1**	**0.040237**	8.1	494
Pallidum, left	**11.8**	**1.8**	**1.2e-10**	**9.0**	**1.9**	**2.4e-06**	**2.8**	**0.5**	**1.4e-07**	24.1	247
Pallidum, right	**13.8**	**1.8**	**5.8e-18**	**12.8**	**1.9**	**4.2e-11**	**3.3**	**0.5**	**1.6e-09**	20.8	247
Putamen, left	**20.4**	**4.0**	**3.2e-07**	**15.5**	**4.1**	**0.00018**	**4.9**	**1.2**	**0.000031**	24.0	608
Putamen, right	**19.4**	**3.9**	**6.1e-07**	**14.4**	**4.0**	**0.00036**	**5.0**	**1.1**	**0.000013**	25.8	590
Thalamus, left	**41.6**	**4.6**	**9.6e-20**	**30.6**	**4.7**	**1.0e-10**	**11.0**	**1.4**	**2.0e-15**	26.4	760
Thalamus, right	**39.4**	**4.3**	**6.7e-20**	**29.5**	**4.5**	**4.5e-11**	**10.0**	**1.3**	**1.8e-14**	25.3	741

Bolded values represent statistically significant estimates at type I error of 0.05

*AD*, Alzheimer’s disease; *ALCOHOL*, alcohol consumption *z*-score; *COGN*, poor cognitive performance *z*-score; *DIET*, diet quality *z*-score; *GM*, gray matter; *HDI*, Healthy Diet Index; *HEALTH*, poor cardio-metabolic and general health *z*-score; *ICV*, intracranial volume; *PA*, physical activity *z*-score; *PRS*, polygenic risk score; *NUTR*, nutritional biomarker *z*-score; *SD*, standard deviation; *SES*, socio-economic status *z*-score; *SEM*, structural equation models; *SMOKING*, smoking *z*-score; *sMRI*, structural magnetic resonance imaging; *SS*, social support *z*-score; *WM*, white Matter; *WMH*, white matter hyperintensity

^a^Values are total, indirect, and direct effects of SES with their associated standard errors and *p*-values, percent of total effect that is mediated and standard deviation value of each outcome. SEM models used are summarized in Supplementary Fig. 1. Selected numerical findings of key path coefficients are presented in Supplementary Table 2 and 3 and illustrated in Fig. 3. Standardized total, indirect and direct effects are further presented in Fig. 2 (heatmap)

^b^See “Methods” section for a full list of exogenous variables entered into the SEM model. ICV was only included for subcortical structures

Table 3 presents TE, DE, and IE of race/ethnicity on the main sMRI outcomes of interest, including total, WM, GM, frontal GM (L/R), and subcortical volumes, and WMH as % ICV (Log_e_-transformed). Non-White vs. White was associated with smaller volumes in general with the exception of WMH which was larger among non-White adults. Total effects were statistically significant for most volumes, with the exception of a few subcortical volumes, namely left amygdala, left caudate, right caudate, left hippocampus, right hippocampus, and left pallidum. For most subcortical volumes, the dominant pattern was a significant total effect of race coupled with significant IE and DE. Nevertheless, complete mediation, with significant TE and IE coupled with non-significant DE, was found for left accumbens, right amygdala, and left putamen. Those findings are summarized in Fig. 2 on standard brain images for subcortical volumes and heat maps for all volumes (RACE_ETHN→sMRI), showcasing the direction and magnitude of standardized effect sizes for TE, IE, and DE. Over 20% of TE of race/ethnicity on WMH was explained by the IE, by a combination of different pathways going through SES, lifestyle, health-related, and cognition factors. This is in contrast to < 10% for the total brain, GM, WM, and frontal GM L/R. For sub-cortical structural volumes and for partial or complete mediation with statistically significant TE and IE going in the same direction (i.e., smaller volumes among non-White adults vs. White adults, after ICV and other exogenous covariate adjustment), % mediation ranged from 9 to 34%.

Table 4 displays the TE, DE, and IE of SES on the primary sMRI outcomes of interest. All the TEs were statistically significant and could be broken down into statistically significant direct effects (DEs) and indirect effects (IEs). There was a positive association between higher SES and larger total, cortical, and subcortical brain volumes. Additionally, individuals with higher SES tend to have a lower volume of WMH relative to their total ICV, after applying a logarithmic transformation. A significant finding is that around 57% of the total effect for SES and the normalized WMH was attributed to an indirect effect. This effect encompasses many pathways that involve lifestyle, health-related, and cognitive aspects. Aside from WMH, the percentage of TE of SES mediated through various pathways ranged from ~ 5% for WM to > 15% up to 36% for most of the remaining sMRI outcomes, which are composed mainly of GM phenotypes. These findings are also visualized in Fig. 2 on standard brain images and the SES-sMRI heat map.

Supplementary Tables 2 and 3 present the detailed results of the SEM models for 4 key sMRI outcomes: total brain volume (TBV), WMH volume as % of ICV (Log_e_-transformed values) or WMH, and bi-lateral hippocampal volumes. Those results are more easily visualized in Fig. 3. *Pathway A* (RACE_ETHN → SES → sMRI) pertained to all 4 outcomes, whereby non-White adults had worse outcomes compared to their White counterparts, with a TE that was at least partially mediated through SES. While the race contrast had a direct effect on TBV and WMH, this was not the case for hippocampal volumes, whereby the total and direct effects were not statistically significant, while the indirect effect had a net inverse association with hippocampal volumes. The main pathways involved in racial/ethnic disparities in sMRI outcomes based on these models were as follows: *Pathway A* (RACE_ETHN( −)→SES(+ / −)→L/R Hippocampus( +), TBV( +) and WMH( −)); *Pathway B* (RACE_ETHN( −)→SES( −)→SMOKING(− / +)→L/R Hippocampus ( −), TBV( −), WMH( +); RACE_ETHN( −)→SES( +)→NUTR( +)→L/R Hippocampus), *Pathway C* (RACE_ETHN( −)→SES( −)→SMOKING( +)→HEALTH(− / +)→L/R Hippocampus ( −), TBV ( −), WMH( +); RACE_ETHN( −)→SES( +)→DIET/SS/NUTR( −)→HEALTH(− / +)→L/R Hippocampus ( −), TBV( −), WMH( +)), *Pathway D* (RACE_ETHN( −)→SES( −)→PA( −)→HEALTH( −)→COGN( −)→L/R Hippocampus( −) and TBV( −)), *Pathway E* (RACE_ETHN ( −)→SES( −)→COGN( −)→L/R Hippocampus ( −) and TBV ( −)), *Pathway F* (RACE_ETHN ( −)→SES ( +)→SS ( −)→COGN( −)→L/R Hippocampus ( −) and TBV ( −)). A number of pathways had net indirect effects of RACE_ETHN that were positively associated with L/R hippocampal volumes and TBV, including several *Pathway F* patterns going through PA, SMOKING, and DIET, reflecting possible reverse causality between baseline cognitive performance and some of the baseline lifestyle factors. Specifically, perceived poor cognitive performance prior to assessment may have led to behavior change in the direction of better diet quality, less smoking, and more frequent and intense PA. Nevertheless, according to Table 5, which examined 6 specific indirect effects across selected sMRI outcomes, the most dominant indirect pathway between race/ethnicity and those selected outcomes was through SES only, constituting 39–57% of the indirect effect. Hypothesized pathway D accounted for < 1% of the indirect effect. For WMH outcome, 16% of the indirect effect was accounted by *pathways B* and *C* (7–9% each). Most of the path coefficients had an associated *p* < 0.001 with few exceptions (Supplementary Tables 2 and 3). Thus, upon correction for multiple testing, most pathways through specific lifestyle factors that were deemed statistically significant at a threshold of 0.05 remained statistically significant at the new threshold of 0.001.

**Table 5:** Racial disparities in selected sMRI volumetric outcomes: six indirect effects through lifestyle, health, and cognition factors: the UK Biobank 2006–2021

	Unstandardized			Percent-mediated		SD	TE	IE
	*β*	*SE*	*p*	*% of TE*	*% of IE*			
***Brain volumes, mm***^***3***^								
**Total brain volume**						**111,279**	** − 61,442**	** − 5169**
RACE_ETHN→SES→sMRI	** − 2528**	**287**	** < 0.001**	**4.1**	**48.9**			
RACE_ETHN→SES→LIFESTYLE→sMRI	** − 161**	**40**	** < 0.001**	0.26	**3.1**			
RACE_ETHN→SES→LIFESTYLE→HEALTH→sMRI	** − 11**	**4**	**0.009**	0.02	0.21			
RACE_ETHN→SES→LIFESTYLE→HEALTH→ COGN→ sMRI	**1.2**	**0.3**	** < 0.001**	− 0.002	− 0.02			
RACE_ETHN→SES→LIFESTYLE → COGN→ sMRI	2	2	0.32	− 0.003	− 0.038			
RACE_ETHN→SES→ COGN→ sMRI	** − 90**	**15**	** < 0.001**	0.146	**1.74**			
**White matter hyperintensity, WMH, % ICV, Log**_**e**_**-transformed**						**0.951**	**0.071**	**0.015**
RACE_ETHN→SES→sMRI	**0.0058**	**0.0016**	** < 0.001**	**8.2**	**39**			
RACE_ETHN→SES→LIFESTYLE→sMRI	**0.0013**	**0.0004**	** < 0.001**	**1.8**	**8.7**			
RACE_ETHN→SES→LIFESTYLE→HEALTH→sMRI	**0.0011**	**0.00013**	** < 0.001**	**1.5**	**7.3**			
RACE_ETHN→SES→LIFESTYLE→HEALTH→ COGN→ sMRI	− 1.1e − 06	1.13e − 06	0.35	− 0.001	− 0.007			
RACE_ETHN→SES→LIFESTYLE → COGN→ sMRI	− 1.80 e − 06	2.6 e − 06	0.49	0	0			
RACE_ETHN→SES→ COGN→ sMRI	0.00008	0.00009	0.33	0.11	0.53			
**Hippocampus, left**						**476**	** − 26**	** − 13**
RACE_ETHN→SES→sMRI	** − 5.0**	**0.9**	** < 0.001**	**19.2**	**38.5**			
RACE_ETHN→SES→LIFESTYLE→sMRI	0.070	0.185	0.71	− 0.27	− 0.54			
RACE_ETHN→SES→LIFESTYLE→HEALTH→sMRI	** − 0.098**	**0.024**	** < 0.001**	0.38	0.75			
RACE_ETHN→SES→LIFESTYLE→HEALTH→ COGN→ sMRI	** + 0.0027**	**0.0008**	**0.001**	− 0.010	− 0.02			
RACE_ETHN→SES→LIFESTYLE → COGN→ sMRI	0.0047	0.0048	0.33	− 0.018	− 0.036			
RACE_ETHN→SES→ COGN→sMRI	** − 0.210**	**0.052**	** < 0.001**	0.81	**1.62**			
**Hippocampus, right**						**494**	** − 25**	** − 9**
RACE_ETHN→SES→sMRI	** − 5.1**	**0.9**	** < 0.001**	**20.4**	**56.7**			
RACE_ETHN→SES→LIFESTYLE→sMRI	− 0.006	0.188	0.98	0.024	0.067			
RACE_ETHN→SES→LIFESTYLE→HEALTH→sMRI	** − 0.054**	**0.022**	**0.014**	0.21	0.6			
RACE_ETHN→SES→LIFESTYLE→HEALTH→ COGN→ sMRI	** + 0.002**	**0.0007**	**0.011**	− 0.008	− 0.02			
RACE_ETHN→SES→LIFESTYLE → COGN→ sMRI	+ 0.003	0.003	0,34	− 0.012	− 0.033			
RACE_ETHN→SES→ COGN→ sMRI	** − 0.1375**	**0.0486**	**0.005**	0.55	**1.53**			

Bolded values represent statistically significant estimates at type I error of 0.05

AD, Alzheimer’s disease; ALCOHOL, alcohol consumption *z*-score; COGN, poor cognitive performance *z*-score; DIET, diet quality *z*-score; GM, gray matter; HDI, Healthy Diet Index; HEALTH, poor cardio-metabolic and general health *z*-score; ICV, intracranial volume; PA, physical activity *z*-score; PRS, polygenic risk score; NUTR, nutritional biomarker *z*-score; SD, standard deviation; SEM, structural equation models; SES, socio-economic status *z*-score; SMOKING, smoking *z*-score; sMRI, structural magnetic resonance imaging; SS, social support *z*-score; WM, white matter; WMH, white matter hyperintensity

^a^Values are six indirect effects of race with their associated standard errors and *p*-values, percent of total effect that is mediated, percent of indirect effect that is mediated, standard deviation value of each outcome, total and indirect effects from SEM. SEM models used are summarized in Supplementary Fig. 1. Selected numerical findings of key path coefficients are presented in Supplementary Table 2 and 3 and illustrated in Fig. 3. Standardized total, indirect, and direct effects are further presented in Fig. 2 (heatmap)

^b^See “Methods” section for a full list of exogenous variables entered into the SEM model. ICV was only included for subcortical structures

## Discussion

This is to our knowledge one of the very few studies to examine mediating pathways between race/ethnicity, SES, and brain sMRI volumetric outcomes. Data from 36,184 UK Biobank participants aged 40–70 years at baseline assessment were used (47% men). We conducted the present study to investigate the differences in sMRI brain markers between racial/ethnic groups (non-White minorities vs. White adults) and socioeconomic disparities. Additionally, we examined the total, direct, and indirect effects of race/ethnicity through lifestyle, health-related factors, and cognition using a structural equations modeling approach. Race/ethnicity, particularly non-White vs. White, and lower SES predicted poorer brain sMRI volumetric outcomes at follow-up. Mediational patterns differ across outcomes, with SES-sMRI total effect partially mediated for all outcomes.

Results of the present study are consistent with findings from multiple recent investigations demonstrating the critical impact of education and other socio-economic factors on brain structure [9, 11, 12, 13, 14, 15, 16, 17, 18, 19, 20, 21, 22, 41, 42]. In a recent review of the relations of SES with global brain volumes and cognitive ability in nearly 500,000 individuals ages 4 to 97 years, 54,000 with brain imaging, from European and US cohorts [22], higher levels of education were associated with greater ICV and total GM volume, whereas higher income was related to greater ICV, but not GM. These associations were stronger in US cohorts versus European cohorts and did not differ by age. Also, similar to our findings, results of analyses from a multi-ethnic cohort (MESA study) found that relations of race and ethnicity with WM lesions were nonsignificant after accounting for SES and cardiovascular risk factors [13].

In another longitudinal study of middle-aged adults, older adults (> / = 65 years) without a college degree exhibited a pattern of declining large-scale functional brain network organization (resting-state system segregation) that was less evident among their college-educated peers [14], while early life factors, including childhood SES, independently predicted small vessel (i.e., WM) disease in later life, even upon adjustment for adult SES and vascular risk factors [12]. Similarly, another study found that across middle age (35–64 years), lower SES was associated with a reduction in resting-state system segregation and reduced gray matter thickness at midlife, patterns not detected in younger and older adulthood [15]. These associations persisted after adjusting for physical, mental health, and cognitive ability in addition to basic demographics [15].

Results of another investigation indicated that age-related decrements in hippocampal volume, which is known to be directly related to memory processing, were greatest among the less educated [9]. Another study reported a positive association between childhood SES and hippocampal volume, after adjusting for mental ability at age 11, adult SES, gender, and education [42], re-enforcing the established neurodevelopmental finding that the impact of early life conditions on structural brain development remains detectable 50 years later [42]. Here, we found race/ethnicity to be a mere instrument for SES which was the main factor linked to hippocampal volume. Furthermore, the total effect of SES on hippocampal volume, upon adjustment for exogenous variables including ICV, was only partially mediated (8–12%) through upstream factors, indicating that SES’s relation with hippocampal volume was mainly a direct one.

The current study further provided evidence of the mediating effects of lifestyle on the association of SES and brain tissue volumes and WMH. Healthier lifestyles which are associated with lower inflammation have been related to better brain structure and health [43], whereas smoking and alcohol dependence have been shown to decrease hippocampus volume [44] and GM volume [45]. Similar to the findings of Dougherty and colleagues using data from the CARDIA study [16], we found that smoking mediated the association of SES with brain outcomes (*Pathway B*, Fig. 3, Table 5), accounting for 27% of the variance previously attributed to SES. Additionally, the results indicated smoking was an indirect mediator of the path connecting lower SES, via poorer health, to greater WMH and smaller TBV and hippocampal volumes. Our findings were similar to those of Raggi and colleagues which revealed the contribution of smoking to social inequalities in health [46]. It is possible that smoking interventions could potentially reduce the health disparity of low SES on brain volume.

This study found that higher SES was associated with greater L/R hippocampus volumes and that higher diet quality and nutritional biomarkers also mediated the paths connecting health with lower L/R hippocampus, TBV, and greater WMH (*Pathways B* and *C*, Fig. 3, Table 5). In previous studies, Mediterranean-type diet patterns were positively associated with preservation of hippocampal volume and larger TBV, while Western-type diets high in trans-fat were associated with less cerebral volume and smaller hippocampal volume [47, 48, 49, 50, 51, 52, 53, 54]. In contrast to the Western-type dietary patterns, diets of higher quality contain nutrients with anti-inflammatory and antioxidant effects [54]. Research using nutrient-based dietary patterns has similarly shown that patterns defined by the antioxidant vitamins C and E, the B vitamins, and vitamin D were associated with greater TBV and less WMH volume, while Western-type diets high in trans-fat were associated with less cerebral volume [55] and smaller hippocampal volume [56].

This study found that physical inactivity was associated with poorer health and cognition in the path connecting SES with L/R hippocampus and TBV (*Pathway E*, Fig. 3, Table 5). Unlike the results reported by Franchetti and colleagues in which lower physical activity was associated with greater WMH in healthy older adults [57], no significant findings were found for WMH in our study. Raja and colleagues reported that while total physical activity was positively associated with L hippocampal volume, vigorous activity was negatively related [58]. Importantly, it has also been found that poorer brain structure is a potential risk factor for physical inactivity [59].

Social support also mediated the association of SES with L/R hippocampus, TBV, and WMH via health (*Pathway C*), as well as the path connecting SES with L/R hippocampus and TBV via cognition (*Pathway F*). This finding is consistent with prior literature demonstrating that social activity was associated with better global cognition [60] and TBV [10]. The importance of social relationships should not be underestimated since their impact may be comparable to physical activity and has been linked to lower inflammation [61, 62]. Community disadvantage in prior research was linked to reduced cortical tissue volume, surface area, and thickness via cardiometabolic and neuroendocrine pathways [17].

With respect to cognition, in a sample of 92 middle-aged Brazilian participants, higher socioeconomic standing was related to better visuospatial ability, executive function, global cognition, and larger parietal lobe volume [11]. The relation between socioeconomic standing and cognitive performance was not mediated by WMH [11]. The latter finding is comparable to ours whereby SES predicted both better cognition and reduced WMH, though there was no pathway between SES and follow-up WMH through poorer baseline cognition.

Our study found that racial differences in frontal GM volumes were partially mediated through SES and other downstream factors, with 5% of the total effect being an indirect effect. Approximately 15% of the total SES effect was mediated by downstream lifestyle, health-related, and cognition factors. A study of middle-aged adults living in Baltimore city, based on the HANDLS SCAN ancillary cohort, reported a mediational pathway between SES and a measure of executive functioning (Trail Making Test Part B) through dorsolateral prefrontal cortex volume for White, but not Black adults, although this was not the case for the Digits Span Forward and verbal fluency tests which were also directly associated with SES [20]. In a comparable study using the same HANDLS SCAN cohort, low SES was linked to greater WM pathology and potentially increased stroke risk in African American adults [21]. In our study, race/ethnicity had a direct and indirect effect through SES and downstream factors, resulting in reduced TBV, GM, WM, and frontal GM volumes and increased WMH as % ICV.

This study is the largest study to examine racial and SES differences in brain volumetric outcomes, using a large array of brain MRI phenotypes to test a model that included a hypothesized pathway and other indirect pathways between race/ethnicity, SES, and brain volumes.

Our study has several limitations. Those include selection bias, measurement error, and residual confounding affect. Moreover, there is not enough statistical power to investigate each racial or ethnic minority independently. Similar pathways that incorporate socioeconomic status (SES) and lifestyle elements like diet quality and exercise were discovered in a parallel study conducted among US and UK older adults with the final outcome being the incidence of dementia, as well as dMRI metrics such as fractional anisotropy [8, 25, 63]. These studies also support the role played by social support in this process [8, 25, 63]. Nevertheless, in our present study, reverse causality is possible in certain models, whereby perceived poor cognitive performance can potentially modify health behaviors which in turn are associated with the final outcome of interest. In fact, although no direct validation study across racial/ethnic groups was done on the cognitive tests included in our present study, it is likely that cultural and social bias exists in the performance particularly when measured only once at a specific point in time. Thus, a longitudinal analysis with multiple repeats may be more appropriate in testing differences across racial groups in terms of cognitive performance change over time.

In sum, our study identified racial minority and lower SES as important determinants of brain volumetric outcomes, with partial mediation of racial/ethnic disparities through SES, lifestyle, health-related, and cognition factors observed for key outcomes. Future longitudinal studies are needed to examine those disparities in a time-dependent manner with multiple repeats on brain volumetric outcomes. Interventions focused on healthy lifestyles for the aging brain could reduce health inequity especially if targeted at high-risk individuals, namely non-White older adults, and people with lower SES.

## Supplementary Information

Below is the link to the electronic supplementary material.

Supplementary file1 (PDF 246 KB)

Supplementary file2 (PDF 138 KB)

## Data Availability

These data are subject to the following licenses and restrictions: UK Biobank is a large-scale biomedical database and research resource containing in-depth genetic and health information from about 500,000 UK participants. The data are augmented regularly with additional records and are globally accessible to approved researchers undertaking vital research into the most common and life-threatening diseases. Requests to access these datasets should be directed to https://www.ukbiobank.ac.uk/.

## Abbreviations

Aβ: Amyloid beta
AD: Alzheimer’s disease
AMICO: Accelerated microstructure imaging via convex optimization
*APOE*: Apolipoprotein E gene
CAPI: Computer-assisted personal interview
CNS: Central nervous system
COGN: Poor cognitive performance *z*-score
CSF: Cerebrospinal fluid
CVD: Cardiovascular disease
CVH: Cardiovascular health
dMRI: Diffusion magnetic resonance imaging
DE: Direct effect
DIET: Diet quality *z*-score
DLPFC: Dorsolateral prefrontal cortex
DTI: Diffusion tensor imaging
DWI: Diffusion-weighted imaging
FA: Fractional anisotropy
FIRST: FMRIB’s integrated registration and segmentation tool
FLAIR: Fluid-attenuated inversion recovery
FoV: Field of view
GM: Gray matter
GWAS: Genome-wide association studies
HANDLS: Healthy aging in neighborhoods of diversity across the life span
HDS: Healthy diet score
HEALTH: Poor health *z*-score
ICD-10: International Classification of Diseases, 10th revision
ICV: Intracranial volume
IDP: Imaging derived phenotype
IE: Indirect effect
LE8: Life’s Essential 8
LS7: Life’s Simple 7
MD: Mean diffusivity
MNI152: Montreal Neurological Institute template with 152 structural images
MPRAGE: Magnetization-prepared 180 degrees radio-frequency pulses and rapid gradient-echo
MRI: Magnetic resonance imaging
NFT: Neurofibrillary tangles
NHB: Non-Hispanic Black
NHW: Non-Hispanic White
NUTR: Nutritional biomarker *z*-score
OLS: Ordinary least square
PA: Physical activity *z-*score
PRS: Polygenic risk score
QC: Quality control
SES: Socioeconomic status
sMRI: Structural magnetic resonance imaging
SS: Social support *z*-score
T1: T1-weighted MRI scans
T2: T2-weighted MRI scans
TBSS: Tract-based spatial statistics
TDI: Townsend Deprivation Index
TE: Total effect
TR: Trace
UK: United Kingdom
WM: White matter
WMH: White matter hyperintensity
WMI: White matter integrity

## Code availability

All codes and related results datasets will be made available on github at baydounm/UKB-paper5-RaceSESsMRI (github.com).

## Declarations

### Disclaimer

The views expressed in this article are those of the authors and do not necessarily reflect the official policy or position of Fort Belvoir Community Hospital, the Defense Health Agency, the Department of Defense, or the U.S. Government. Reference to any commercial products within this publication does not create or imply any endorsement by Fort Belvoir Community Hospital, the Defense Health Agency, the Department of Defense, or the U.S. Government.
