# Supplementary material for: Uncovering mediational pathways behind racial and socioeconomic disparities in brain volumes: insights from the UK Biobank study
Source: GeroScience. 2024 Oct 10;47(2):1837–58. doi: 10.1007/s11357-024-01371-1 (PMC11979012; doi:10.1007/s11357-024-01371-1)
Supplement: Supplementary file 1 — Supplementary file1 (PDF 246 KB) [file 11357_2024_1371_MOESM1_ESM.pdf]

ONLINE SUPPLEMENTARY MATERIALS

Supplementary Method 1: Brain imaging derived phenotypes or IDPs and detailed processing

Main Source: http://biobank.ctsu.ox.ac.uk/crystal/docs/brain_mri.pdf

sMRI processing

The sMRI processing procedure encompasses the analyses of T1, T2, and T2 FLAIR images. Following deidentification and bias field reduction, brain-extracted T1 images are obtained following nonlinear warping of data using the MN1152 space, and back-transformation with a standard space brain mask. The FAST algorithm is employed for the segmentation of brain tissue, namely cerebrospinal fluid (CSF), grey matter, and white matter, as well as their corresponding partial-volume images. A Structural Image Evaluation (SIENAX)-style analysis is conducted to provide segmented tissue and total brain volumes as Image Data Points (IDPs). An extra 139 imaging-defined phenotypes (IDPs) are generated by summing the estimates of grey matter partial volume. FreeSurfer is exclusively utilized for the processing of T1 images, whereas T2_FLAIR is employed in conjunction with T1 images wherever feasible. The last stage is quality control (QC) of the FreeSurfer outputs, employing the Qoala-T methodology. This T2 FLAIR processing entails matching the T2 picture linearly with the T1 image and converting it directly into the T1 space and MNI standard space of the particular patient. White matter lesions are segmented automatically using the BIANCA tool to determine the overall white matter hyperintensity (WMH) volume, therefore producing an extra intradural pressure (IDP).

Sources:

Main source: http://biobank.ctsu.ox.ac.uk/crystal/docs/brain_mri.pdf

Other sources: (1, 2, 3, 4, 5, 6, 7, 8, 9)

Supplementary Table 1. Detailed description and definition of sMRI measures used

sMRI									
**Measure**	**Left/Right (yes/no)**	**UKB field**		**ROI abbrev.**	**Region**	**GM/WM/Both**	**Cortical (y/n)**	**Tract (y/n)**	**Sub-cortical (y/n)**
**Brain volumes**									
**Intracranial volume, ICV**		26521		**ICV**	**Total brain**	**Both**	**Y**	**Y**	**Y**
**White Matter Hyperintensity, WMH**	**No**	25781		**WMH**	**Total brain**	**WM**	**Y**	**N**	**Y**
Total	***No***	25010		***TOTALBRAIN***	***Total brain***	***Both***	***Y***	***N***	***Y***
Total GM	***No***	25006		***GM***	***Gray matter***	***GM***	***Y***	***N***	***Y***
Total WM	***No***	25008		***WM***	***White Matter***	***WM***	***Y***	***Y***	***Y***
FGM	***Yes***	Sum below, Left/Right		***Left_Frontal_GM*** ***Right_Frontal_GM***	***Frontal Gray Matter***	***GM***	***Y***	***N***	***N***
Frontal medial cortex	Yes	25830 and 25831		Left_Frontal_MC Right_Frontal_MC	Frontal Gray Matter	GM	Y	N	N
Frontal Operculum	Yes	25862 and 25863		Left_Frontal_OP Right_Frontal_OP	Frontal Gray Matter	GM	Y	N	N
Frontal Orbital Cortex	Yes	25846 and 25847		Left_Frontal_OC Right_Frontal_OC	Frontal Gray Matter	GM	Y	N	N
Frontal Pole	Yes	25782 and 25783		Left_Frontal_pole Right_Frontal_pole	Frontal Gray Matter	GM	Y	N	N
Inferior Frontal Gyrus, pars opercularis	Yes	25792 and 25793		Left_IFG_PO Right_IFG_PO	Frontal Gray Matter	GM	Y	N	N

Inferior Frontal Gyrus, pars triangularis	Yes	25790 and 25791		Left_IFG_PT<br><br>Right_IFG_PT	Frontal Gray Matter	GM	Y	N	N
Juxtapositional Lobule Cortex	Yes	25832 and 25833		Left_JXLC<br><br>Right_JXLC	Frontal Gray Matter	GM	Y	N	N
Middle Frontal Gyrus	Yes	25788 and 25789		Left_MFG<br><br>Right_MFG	Frontal Gray Matter	GM	Y	N	N
Precentral Gyrus	Yes	25794 and 25795		Left_PG<br><br>Right_PG	Frontal Gray Matter	GM	Y	N	N
Superior Frontal Gyrus	Yes	25786 and 25787		Left_SFG<br><br>Right_SFG	Frontal Gray Matter	GM	Y	N	N
Sub-cortical volumes									
Accumbens	Yes	25023 and 25024		Left_Accumbens<br><br>Right_Accumbens	Sub-cortical volumes	GM	N	N	Y
Amygdala	Yes	25021 and 25022		Left_Amygdala<br><br>Right_Amygdala	Sub-cortical volumes	GM	N	N	Y
Caudate	Yes	25013 and 25014		Left_Caudate<br><br>Right_Caudate	Sub-cortical volumes	GM	N	N	Y
Hippocampus	Yes	25019 and 25020		Left_Hippocampus<br><br>Right_Hippocampus<br>N	Sub-cortical volumes	GM	N	N	Y
Pallidum	Yes	25017 and 25018		Left_Pallidum<br><br>Right_Pallidum	Sub-cortical volumes	GM	N	N	Y
Putamen	Yes	25015 and 25016		Left_Putamen<br><br>Right_Putamen	Sub-cortical volumes	GM	N	N	Y
Thalamus	Yes	25011 and 25012		Left_Thalamus<br><br>Right_Thalamus	Sub-cortical volumes	GM	N	N	Y

Supplemental method 2: Mediator and other covariate operationalization(10, 11, 12)

The primary investigation of the UK Biobank utilized a touchscreen questionnaire to evaluate the standard of one's dietary intake. The survey inquired on the frequency of food consumption and the characteristics of different food categories, including cooked vegetables, salad/raw veggies, fresh fruit, and processed meats. The survey also inquired about the variety of milk, spread, bread, morning cereal, coffee, and any dietary restrictions. The validity of the questionnaire was assessed by comparing it to 24-hour recall data obtained from participants in the UK Biobank study. The results indicated that there was sufficient agreement in the ranking of each food group of interest. The questionnaire was revised to accurately incorporate the data accessibility within the UK biobank. Details are provided elsewhere(10, 11, 12), along with criteria used to construct the Healthy Diet score. Furthermore, Stata code can be made available upon request.

Three tobacco exposure variables were generated using a touchscreen questionnaire at the assessment center visit, including smoking status, environmental tobacco smoke, and pack-years of smoking. Alcohol consumption was assessed through quantity-frequency questions, with the construct ALCOHOL being the standardized z-score for this item. Physical activity was measured using self-reported responses, resulting in MET.min/week for each category of physical activity intensity. Diet quality was measured using dietary recommendations, and nutritional biomarkers like Vitamin D and red cell distribution width (RDW) were selected as additional markers.

Social support was evaluated using three variables: how often do you visit friends or family, how often are you able to confide in someone close to you, and which of the following do you attend once a week or more often? These measures were then transformed into a standardized z-score and averaged into the SS measure.

The general and cardio-metabolic health construct combined body mass index (BMI), allostatic load (AL), a co-morbidity index, and self-rated health. BMI was computed at baseline assessment, while AL total score was an index that adds up with equal weighting of cardiovascular, metabolic, and inflammatory risk indicators. Co-morbidity index was constructed using two data fields based on self-reported data on pre-existing co-morbidities. Self-rated health was obtained as part of the touchscreen questionnaire at baseline assessment.

Cognitive performance and poor cognitive performance score (COGN) were assessed using touchscreen computer assessments. The study focused on the 6 pair version of the visual memory task due to its greater difficulty and the time to complete it. Poor cognitive performance scores were obtained from three cognitive test scores, with a higher score indicating poorer cognitive performance.

The study used a Bayesian approach to generate PRS scores and apply them to meta-analyzed summary statistics GWAS data from external and internal UK Biobank data. The Standard PRS

Set included 28 diseases and 8 quantitative traits. Details on how AD PRS was computed is provided elsewhere(10, 11, 12).

Blood biochemistry was assessed at baseline, including markers for liver and kidney function, systemic inflammation, lipid metabolism, glucose homeostasis, and calcium metabolism. Risk indicators included albumin, C-reactive protein, total cholesterol, HDL-cholesterol, and glycated hemoglobin. Blood measures included total cholesterol, HDL-cholesterol, CRP, albumin, and glycosylated hemoglobin. Blood lipids were measured using direct enzymatic methods. Other measures included waist-to-hip ratio, radial pulse, and systolic and diastolic blood pressure. Details on how the allostatic load was constructed, and criteria for computing the total score is detailed elsewhere(10, 11, 12).

Supplementary Table 2. Pathways from race/ethnicity (Non-White vs. White) to selected sMRI volumetric (total brain volume and WMH as % ICV, Log_e transformed) outcomes through modifiable risk factors among UK biobank participants, 2006-2021 [a]

	TBV		WMH, as % ICV, Log_e transformed	
	β	(SE), p	β	(SE), p
Main pathway				
RACE_ETHN→SES (β_{12})	**-0.203**	**(0.020), p<0.001**	**-0.203**	**(0.020), p<0.001**
SES→SS (β_{23})	**+0.042**	**(0.005), p<0.001**	**+0.042**	**(0.005), p<0.001**
SES→PA(β_{24})	**-0.126**	**(0.009), p<0.001**	**-0.128**	**(0.009), p<0.001**
SES→DIET(β_{25})	**+0.136**	**(0.009), p<0.001**	**+0.136**	**(0.009), p<0.001**
SES → NUTR (β_{26})	**+0.0181**	**(0.006), p<0.001**	**+0.0181**	**(0.006), p<0.001**
SES → SMOKING (β_{27})	**-0.126**	**(0.006), p<0.001**	**-0.126**	**(0.006), p<0.001**
SES → ALCOHOL (β_{28})	**+0.262**	**(0.008), p<0.001**	**+0.262**	**(0.008), p<0.001**
SS → HEALTH (β_{39})	**-0.136**	**(0.006), p<0.001**	**-0.136**	**(0.006), p<0.001**
PA → HEALTH (β_{49})	**-0.067**	**(0.004), p<0.001**	**-0.067**	**(0.004), p<0.001**
DIET → HEALTH (β_{59})	**-0.066**	**(0.003), p<0.001**	**-0.066**	**(0.003), p<0.001**
NUTR → HEALTH (β_{69})	**-0.094**	**(0.005), p<0.001**	**-0.094**	**(0.005), p<0.001**
SMOKING → HEALTH (β_{79})	**+0.067**	**(0.005), p<0.001**	**+0.067**	**(0.005), p<0.001**
ALCOHOL → HEALTH (β_{89})	**-0.059**	**(0.003), p<0.001**	**-0.059**	**(0.003), p<0.001**
HEALTH→COGN (β_{910})	**-0.052**	**(0.010), p<0.001**	**-0.052**	**(0.010), p<0.001**
COGN → sMRI (β_{1011})	**-4,024**	**(394), p<0.001**	0.004	(0.004), p=0.33
Selected direct effects on final outcomes				
RACE_ETHN→sMRI (β_{111})	**-61,442**	**(2,735), p<0.001**	**+0.056**	**(0.026), p=0.035**
SES → sMRI (β_{211})	**+1,246**	**(757), p<0.001**	**-0.0288**	**(0.008), p<0.001**
SS → sMRI (β_{311})	**+2,303**	**(754), p<0.001**	0.003	(0.0073), p=0.71
PA → sMRI (β_{411})	+310	(469), p=0.51	***+0.011***	***(0.005), p=0.014***
DIET → sMRI (β_{511})	**+3,467**	**(473), p<0.001**	-0.0069	(0.0046), p=0.13

NUTR → sMRI (β_{611})	-106	(680), p=0.88	-0.0025	(0.007), p=0.70
SMOKING → sMRI (β_{711})	**-2,338**	**(642), p<0.001**	**+0.054**	**(0.006), p<0.001**
ALCOHOL→ sMRI (β_{811})	-78	(485), p=0.87	***+0.009***	***(0.005), p=0.044***
HEALTH → sMRI (β_{911})	**-2,025**	**(750), p=0.007**	**+0.190**	**(0.007), p<0.001**
Other effects between endogenous variables				
SES→HEALTH (β_{29})	**-0.136**	**(0.006), p<0.001**	**-0.136**	**(0.006), p<0.001**
SES→COGN (β_{211})	**-0.111**	**(0.010), p<0.001**	**-0.111**	**(0.010), p<0.001**
SS→COGN (β_{311})	***-0.025***	***(0.010) ,p=0.014***	***-0.025***	***(0.010) ,p=0.014***
PA→COGN (β_{411})	**+0.025**	**(0.006), p<0.001**	**+0.025**	**(0.006), p<0.001**
DIET→COGN (β_{511})	**+0.021**	**(0.006), p=0.001**	**+0.021**	**(0.006), p=0.001**
NUTR→COGN (β_{611})	-0.005	(0.009), p=0.57	-0.005	(0.009), p=0.57
SMOKING→COGN (β_{711})	**-0.055**	**(0.009), p<0.001**	**-0.055**	**(0.009), p<0.001**
ALCOHOL→COGN (β_{811})	-0.011	(0.006), P=0.082	-0.011	(0.006), P=0.082
Other direct effects of race				
RACE_ETHN→SS (β_{13})	**-0.226**	**(0.019), p<0.001**	**-0.226**	**(0.019), p<0.001**
RACE_ETHN→PA(β_{14})	-0.044	(0.031), p=0.15	-0.044	(0.031), p=0.15
RACE_ETHN→DIET(β_{15})	**+0.137**	**(0.030), p<0.001**	**+0.137**	**(0.030), p<0.001**
RACE_ETHN→NUTR(β_{16})	**-0.502**	**(0.022), p<0.001**	**-0.502**	**(0.022), p<0.001**
RACE_ETHN→SMOKING(β_{17})	***-0.060***	***(0.022), p=0.007***	***-0.060***	***(0.022), p=0.007***
RACE_ETHN→ALCOHOL(β_{18})	**-0.698**	**(0.030), p<0.001**	**-0.698**	**(0.030), p<0.001**
RACE_ETHN→HEALTH(β_{19})	-0.033	(0.020), p=0.088	**-0.033**	(**0.020**), p=0.088
RACE_ETHN→COGN(β_{110})	**+0.581**	**(0.036), p<0.001**	**+0.581**	**(0.036) ,p<0.001**

Abbreviations: AD=Alzheimer's Disease; ALCOHOL=Alcohol consumption z-score; COGN=Poor cognitive performance z-score; DIET=Diet quality z-score; HEALTH=Poor cardio-metabolic and general health z-score; ICV=Intracranial volume; PA=Physical Activity z-score; PRS=Polygenic Risk Score; NUTR=Nutritional biomarker z-score; RACE_ETHN=Race/ethnicity contrast: Non-White vs. White; SES=Socio-economic status z-score; SMOKING=Smoking z-score; sMRI=Structural magnetic resonance imaging; SS=Social Support z-score WM=White Matter; WMH=White Matter Hyperintensity.

[a] Values are path coefficients β ± SE or non-linear combinations of path coefficients to compute selected indirect effects, across 5 imputations with 10 iterations. For indirect effects, 1 through 5 represent estimates for each extracted imputation. Rubin's rule refers to pooled estimate across the 5 imputations using Rubin's rule for point estimates and standard errors.

*P<0.05 **P<0.01 ***P<0.001 for null hypothesis of $\beta=0$. Only P<0.001 is bolded, while P>0.001 but <0.05 is bolded and italicized to adjust for multiple testing.

Supplementary Table 3. Pathways from race/ethnicity (Non-White vs. White) to selected sMRI volumetric (Left and Right hippocampal volumes) outcomes through modifiable risk factors among UK biobank participants, 2006-2021 [a]

	Left Hippocampal volume		Right Hippocampal volume	
	β	(SE), p	β	(SE), p
Main pathway				
RACE_ETHN→SES (β_{12})	**-0.203**	**(0.020), p<0.001**	**-0.203**	**(0.020), p<0.001**
SES→SS (β_{23})	**+0.042**	**(0.005), p<0.001**	**+0.042**	**(0.005), p<0.001**
SES→PA(β_{24})	**-0.126**	**(0.009), p<0.001**	**-0.128**	**(0.009), p<0.001**
SES→DIET(β_{25})	**+0.136**	**(0.009), p<0.001**	**+0.136**	**(0.009), p<0.001**
SES → NUTR (β_{26})	**+0.0181**	**(0.006), p<0.001**	**+0.0181**	**(0.006), p<0.001**
SES → SMOKING (β_{27})	**-0.126**	**(0.006), p<0.001**	**-0.126**	**(0.006), p<0.001**
SES → ALCOHOL (β_{28})	**+0.262**	**(0.008), p<0.001**	**+0.262**	**(0.008), p<0.001**
SS → HEALTH (β_{39})	**-0.136**	**(0.006), p<0.001**	**-0.136**	**(0.006), p<0.001**
PA → HEALTH (β_{49})	**-0.067**	**(0.004), p<0.001**	**-0.067**	**(0.004), p<0.001**
DIET → HEALTH (β_{59})	**-0.066**	**(0.003), p<0.001**	**-0.066**	**(0.003), p<0.001**
NUTR → HEALTH (β_{69})	**-0.094**	**(0.005), p<0.001**	**-0.094**	**(0.005), p<0.001**
SMOKING → HEALTH (β_{79})	**+0.067**	**(0.005), p<0.001**	**+0.067**	**(0.005), p<0.001**
ALCOHOL → HEALTH (β_{89})	**-0.059**	**(0.003), p<0.001**	**-0.059**	**(0.003), p<0.001**
HEALTH→COGN (β_{910})	**-0.052**	**(0.010), p<0.001**	**-0.052**	**(0.010), p<0.001**
COGN → sMRI (β_{1011})	**-9.3**	**(2.0), p<0.001**	***-6.1***	***(2.0), p=0.010***
Selected direct effects on final outcomes				
RACE_ETHN→sMRI (β_{111})	-13.5	(13.8), p=0.33	-15.4	(14.0), p=0.27
SES → sMRI (β_{211})	**+24.7**	**(3.9), p<0.001**	**+ 25.2**	**(4.0), p<0.001**
SS → sMRI (β_{311})	+3.7	(3.8), p=0.33	1.9	(3.8), p=0.63
PA → sMRI (β_{411})	2.3	(2.4), p=0.33	1.0	(2.4), p=0.67
DIET → sMRI (β_{511})	-1.3	(2.4), p=0.58	1.9	(2.4), p=0.43
NUTR → sMRI (β_{611})	**+8.2**	**(3.5), p=0.018**	**+8.9**	**(3.4), p=0.010**
SMOKING → sMRI (β_{711})	**-10.5**	**(3.2), p=0.001**	**-10.4**	**(3.3), p=0.002**

ALCOHOL→ sMRI (β_{811})	-0.011	(0.006), p=0.082	***-6.3***	***(2.5), p=0.010***
HEALTH → sMRI (β_{911})	**-17.7**	**(3.8), p<0.001**	**-9.6**	**(3.8), p=0.011**
Other effects between endogenous variables				
SES→HEALTH (β_{29})	**-0.136**	**(0.006), p<0.001**	**-0.136**	**(0.006), p<0.001**
SES→COGN (β_{211})	**-0.111**	**(0.010), p<0.001**	**-0.111**	**(0.010), p<0.001**
SS→COGN (β_{311})	***-0.025***	***(0.010) ,p=0.014***	***-0.025***	***(0.010) ,p=0.014***
PA→COGN (β_{411})	**+0.025**	**(0.006), p<0.001**	**+0.025**	**(0.006), p<0.001**
DIET→COGN (β_{511})	**+0.021**	**(0.006), p=0.001**	**+0.021**	**(0.006), p=0.001**
NUTR→COGN (β_{611})	-0.005	(0.009), p=0.57	-0.005	(0.009), p=0.57
SMOKING→COGN (β_{711})	**-0.055**	**(0.009), p<0.001**	**-0.055**	**(0.009), p<0.001**
ALCOHOL→COGN (β_{811})	-0.011	(0.006), P=0.082	-0.011	(0.006), P=0.082
Other direct effects of race				
RACE_ETHN→SS (β_{13})	**-0.226**	**(0.019), p<0.001**	**-0.226**	**(0.019), p<0.001**
RACE_ETHN→PA(β_{14})	-0.044	(0.031), p=0.15	-0.044	(0.031), p=0.15
RACE_ETHN→DIET(β_{15})	**+0.137**	**(0.030), p<0.001**	**+0.137**	**(0.030), p<0.001**
RACE_ETHN→NUTR(β_{16})	**-0.502**	**(0.022), p<0.001**	**-0.502**	**(0.022), p<0.001**
RACE_ETHN→SMOKING(β_{17})	**-0.060**	**(0.022), p=0.007**	**-0.060**	**(0.022), p=0.007**
RACE_ETHN→ALCOHOL(β_{18})	**-0.698**	**(0.030), p<0.001**	**-0.698**	**(0.030), p<0.001**
RACE_ETHN→HEALTH(β_{19})	-0.033	(0.020), p=0.088	-0.033	(0.020), p=0.088
RACE_ETHN→COGN(β_{110})	**+0.581**	**(0.036), p<0.001**	**+0.581**	**(0.036) ,p<0.001**

Abbreviations: AD=Alzheimer's Disease; ALCOHOL=Alcohol consumption z-score; COGN=Poor cognitive performance z-score; DIET=Diet quality z-score; HEALTH=Poor cardio-metabolic and general health z-score; ICV=Intracranial volume; PA=Physical Activity z-score; PRS=Polygenic Risk Score; NUTR=Nutritional biomarker z-score; RACE_ETHN=Race/ethnicity contrast: Non-White vs. White; SES=Socio-economic status z-score; SMOKING=Smoking z-score; sMRI=Structural magnetic resonance imaging; SS=Social Support z-score.

[a] Values are path coefficients β ± SE or non-linear combinations of path coefficients to compute selected indirect effects, across 5 imputations with 10 iterations. For indirect effects, 1 through 5 represent estimates for each extracted imputation. Rubin's rule refers to pooled estimate across the 5 imputations using Rubin's rule for point estimates and standard errors.

*$P<0.05$ **$P<0.01$ ***$P<0.001$ for null hypothesis of $\beta=0$. Only $P<0.001$ is bolded, while $P>0.001$ but <0.05 is bolded and italicized to adjust for multiple testing.

SUPPLEMENTARY REFERENCES

1. Zhang Y, Brady M, Smith S. Segmentation of brain MR images through a hidden Markov random field model and the expectation-maximization algorithm. IEEE Trans Med Imaging. 2001;20(1):45-57.
2. Fischl B, Salat DH, Busa E, Albert M, Dieterich M, Haselgrove C, et al. Whole brain segmentation: automated labeling of neuroanatomical structures in the human brain. Neuron. 2002;33(3):341-55.
3. Fischl B, Sereno MI, Dale AM. Cortical surface-based analysis. II: Inflation, flattening, and a surface-based coordinate system. Neuroimage. 1999;9(2):195-207.
4. Dale AM, Fischl B, Sereno MI. Cortical surface-based analysis. I. Segmentation and surface reconstruction. Neuroimage. 1999;9(2):179-94.
5. Fischl B, van der Kouwe A, Destrieux C, Halgren E, Segonne F, Salat DH, et al. Automatically parcellating the human cerebral cortex. Cereb Cortex. 2004;14(1):11-22.
6. Desikan RS, Segonne F, Fischl B, Quinn BT, Dickerson BC, Blacker D, et al. An automated labeling system for subdividing the human cerebral cortex on MRI scans into gyral based regions of interest. Neuroimage. 2006;31(3):968-80.
7. Iglesias JE, Augustinack JC, Nguyen K, Player CM, Player A, Wright M, et al. A computational atlas of the hippocampal formation using ex vivo, ultra-high resolution MRI: Application to adaptive segmentation of in vivo MRI. Neuroimage. 2015;115:117-37.
8. Klapwijk ET, van de Kamp F, van der Meulen M, Peters S, Wierenga LM. Qoala-T: A supervised-learning tool for quality control of FreeSurfer segmented MRI data. Neuroimage. 2019;189:116-29.
9. Griffanti L, Zamboni G, Khan A, Li L, Bonifacio G, Sundaresan V, et al. BIANCA (Brain Intensity AbNormality Classification Algorithm): A new tool for automated segmentation of white matter hyperintensities. Neuroimage. 2016;141:191-205.
10. Beydoun MA, Beydoun HA, Fanelli-Kuczmarski MT, Weiss J, Georgescu MF, Meirelles O, et al. Pathways explaining racial/ethnic and socio-economic disparities in dementia incidence: the UK Biobank study. Aging (Albany NY). 2023;15(18):9310-40.
11. Beydoun MA, Beydoun HA, Hu YH, El-Hajj ZW, Georgescu MF, Noren Hooten N, et al. Helicobacter pylori, persistent infection burden and structural brain imaging markers. Brain Commun. 2024;6(2):fcae088.
12. Weiss J, Beydoun MA, Beydoun HA, Georgescu MF, Hu YH, Noren Hooten N, et al. Pathways explaining racial/ethnic and socio-economic disparities in brain white matter integrity outcomes in the UK Biobank study. SSM Popul Health. 2024;26:101655.

SUPPLEMENTARY FIGURE LEGEND

SUPPLEMENTARY FIGURE 1. Conceptual Framework

Abbreviations: ALCOHOL=Alcohol consumption z-score; COGN=Poor cognitive performance z-score; DIET=Diet quality z-score; HEALTH=Poor cardio-metabolic and general health z-score; ICV=Intracranial volume; PA=Physical Activity z-score; NUTR=Nutritional biomarker z-score; SD=Standard Deviation; SEM= Structural Equations Model; SES=Socio-economic status z-score; SMOKING=Smoking z-score; sMRI=Structural magnetic resonance imaging; SS=Social Support z-score; WM=White Matter; WMH=White Matter Hyperintensity.

Note: Figure generated using powerpoint and http://www.biorender.com.
