# Supplementary material for: Uncovering mediational pathways behind racial and socioeconomic disparities in brain volumes: insights from the UK Biobank study
Source: GeroScience. 2024 Oct 10;47(2):1837–58. doi: 10.1007/s11357-024-01371-1 (PMC11979012; doi:10.1007/s11357-024-01371-1)
Supplement: Supplementary file 2 — Supplementary file2 (PDF 138 KB) [file 11357_2024_1371_MOESM2_ESM.pdf]

SUPPLEMENTARY FIGURE 1. Conceptual Framework

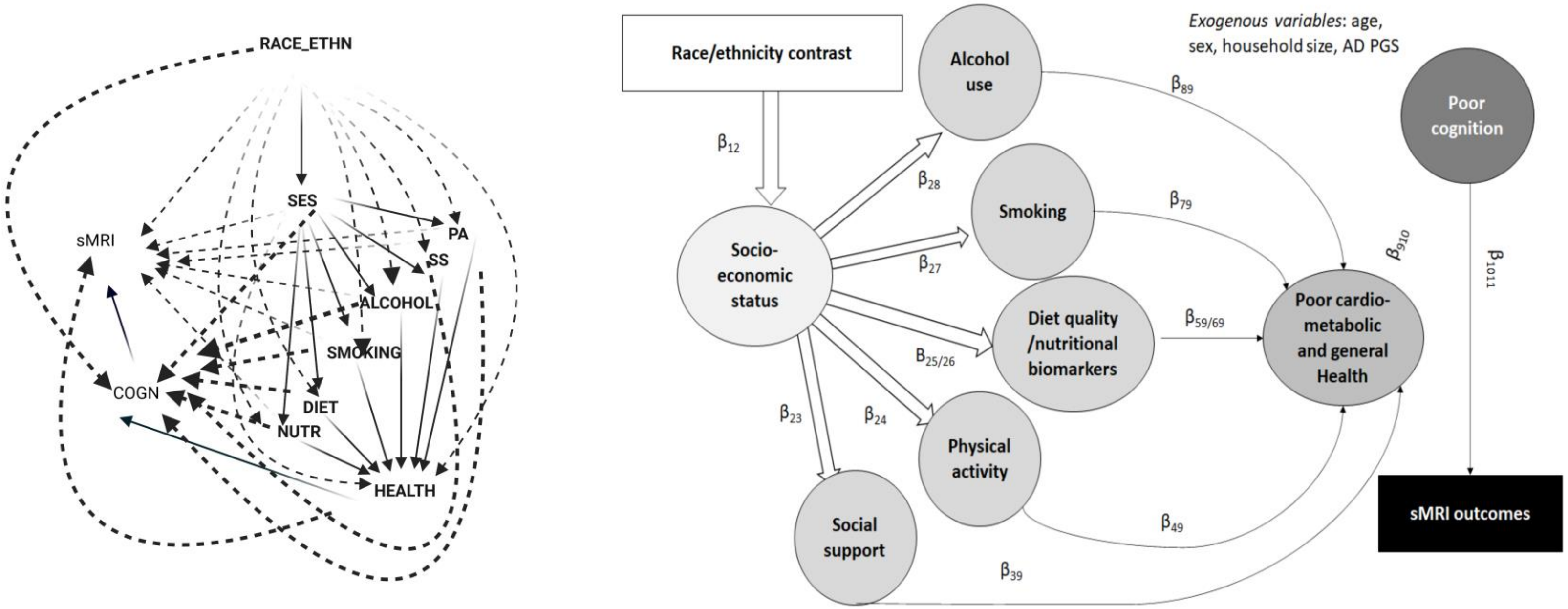

Abbreviations: ALCOHOL=Alcohol consumption z-score; COGN=Poor cognitive performance z-score; DIET=Diet quality z-score; HEALTH=Poor cardio-metabolic and general health z-score; ICV=Intracranial volume; PA=Physical Activity z-score; NUTR=Nutritional biomarker z-score; SD=Standard Deviation; SEM= Structural Equations Model; SES=Socio-economic status z-score; SMOKING=Smoking z-score; sMRI=Structural magnetic resonance imaging; SS=Social Support z-score; WM=White Matter; WMH=White Matter Hyperintensity.

Note: Figure generated using powerpoint and http://www.biorender.com.
